# Immune evasion in lung metastasis of leiomyosarcoma: upregulation of EPCAM inhibits CD8^+^ T cell infiltration

**DOI:** 10.1038/s41416-024-02576-z

**Published:** 2024-01-30

**Authors:** Masaya Kanahori, Eijiro Shimada, Yoshihiro Matsumoto, Makoto Endo, Toshifumi Fujiwara, Akira Nabeshima, Takeshi Hirose, Kengo Kawaguchi, Ryunosuke Oyama, Yoshinao Oda, Yasuharu Nakashima

**Affiliations:** 1 Department of Orthopaedic Surgery, Graduate School of Medical Science, Kyushu University, Fukuoka, Japan; 2 Department of Orthopaedic Surgery, Fukushima Medical University School of Medicine, Fukushima, Japan; 3 Department of Anatomic Pathology, Pathological Sciences, Graduate School of Medical Science, Kyushu University, Fukuoka, Japan

**Keywords:** Sarcoma, Immunoediting, Metastasis, CD8-positive T cells, Immune evasion

## Abstract

**Background:**

Leiomyosarcomas are among the most common histological types of soft tissue sarcoma (STS), with no effective treatment available for advanced patients. Lung metastasis, the most common site of distant metastasis, is the primary prognostic factor. We analysed the immune environment targeting lung metastasis of STS to explore new targets for immunotherapy.

**Methods:**

We analysed the immune environment of primary and lung metastases in 38 patients with STS using immunohistochemistry. Next, we performed gene expression analyses on primary and lung metastatic tissues from six patients with leiomyosarcoma. Using human leiomyosarcoma cell lines, the effects of the identified genes on immune cells were assessed in vitro.

**Results:**

Immunohistochemistry showed a significant decrease in CD8^+^ cells in the lung metastases of leiomyosarcoma. Among the genes upregulated in lung metastases, epithelial cellular adhesion molecule (EPCAM) showed the strongest negative correlation with the number of CD8^+^ cells. Transwell assay results showed that the migration of CD8^+^ T cells was significantly increased in the conditioned media obtained after inhibition or knock down of EPCAM.

**Conclusions:**

EPCAM was upregulated in lung metastases of leiomyosarcoma, suggesting inhibition of CD8^+^ T cell migration. Our findings suggest that EPCAM could serve as a potential novel therapeutic target for leiomyosarcoma.

## Introduction

Soft tissue sarcomas (STS) are rare and heterogeneous mesenchymal neoplasms that account for 1% of all malignant adult tumours and have more than 100 histological subtypes [1]. Leiomyosarcoma (LMS) is one of the most common histological types of STS [2]. However, there is still no effective treatment for patients with distant metastases [3]. The median overall survival is 12–18 months [4].

Immunotherapy has recently emerged as a new treatment modality, in combination with surgery, chemotherapy, and radiotherapy [5]. Immune checkpoint inhibitors have shown promising results against malignant tumours such as melanoma, non-small cell lung cancer, renal cell carcinoma, and head and neck cancer [6–9], whereas numerous other malignant tumours show poor responses, yielding poor results in clinical trials [10]. Clinical trials have reported limited effectiveness for LMS and most STS types, except for some histological types, such as dedifferentiated liposarcoma and undifferentiated pleomorphic sarcoma [11].

Recent studies have reported that in some malignant tumours, the immune environment differs between the primary and metastatic sites [12]. Therefore, the response to immunotherapy may differ between primary and metastatic sites [13]. In LMS, surgical therapy is the first choice for resectable primary sites, and new treatments are expected to be effective for unresectable primary sites and distant metastases [14]. Lung metastasis is the predominant form of distant metastasis and represents a crucial prognostic factor for LMS [15]. Therefore, immunotherapy that targets lung metastases from LMS may be extremely useful. However, almost no research has been conducted on the immune environment of the metastatic sites of STS, including LMS.

This study analysed differences in the immune environment using clinical samples from primary and lung metastatic sites of STS, including LMS. We discovered unique immune evasion in lung metastases of LMS, which supports the results of clinical immunotherapy trials. Therefore, we aimed to elucidate the mechanism of this immune evasion and explore novel treatment targets.

## Materials and methods

### Patient tumour samples

We utilised formalin-fixed, paraffin-embedded STS tissue specimens of STS samples collected at Kyushu University Hospital between 2005 and 2021. Biopsy tissues were excluded, and resected tissues were targeted. Consequently, we used samples from 38 patients with primary tumours and lung metastases. All tissue samples were examined by pathologists. All the patients provided written informed consent before participating in the study. The Institutional Review Board of Kyushu University approved the use of human specimens for this study (approval number: 23005-02). The histological types and clinical features of the target samples are presented in Supplementary Table 1.

### Immunohistochemistry

Immunohistochemical staining was performed on 3 μm thin sections of the paraffin-embedded tissue specimens. The primary antibodies used were as follows: anti-cluster of differentiation (CD)4 (#48274, Cell Signaling Technology, Beverly, MA, USA), anti-CD8α (#70306, Cell Signaling Technology), anti-forkhead box protein 3 (FoxP3) (#98377, Cell Signaling Technology), anti-CD20 (#74332, Cell Signaling Technology), anti-CD56 (#99746, Cell Signaling Technology), anti-CD68 (#76437, Cell Signaling Technology), anti-CD163 (#93498, Cell Signaling Technology), anti-programmed cell death protein 1 (PD-1) (#86163, Cell Signaling Technology), anti-programmed death-ligand 1 (PD-L1) (#13684, Cell Signaling Technology), and anti-human leukocyte antigen (HLA) class I ABC (#ab70328, Abcam, Cambridge, UK). The blocking reagent used was 10% Normal Goat Serum (#426041, Nichirei Biosciences, Tokyo, Japan), and the secondary antibody was EnVision Dual Link System horseradish peroxidase (HRP) (K406311, Dako). Antigen activation was performed via heat-induced antigen retrieval with a 10-mM Na citrate buffer (for anti-CD4, anti-FoxP3, anti-CD20, anti-CD68, anti-CD163, anti-PD-1, and anti-HLA class I ABC), 1 mM ethylenediaminetetraacetic acid (EDTA) (for anti-CD8 and anti-PD-L1), or 10 mM Tris/1 mM EDTA (for anti-CD56). All samples were deparaffinised in xylene and dehydrated using a graded ethanol series. After heat-induced antigen retrieval, endogenous peroxidase blocking and blocking treatments were performed, and the samples were incubated overnight at 4 °C with the primary antibody. After incubation with the secondary antibody, the samples were visualised using a diaminobenzidine substrate system (TCI Chemicals, Tokyo, Japan) and counterstained with diluted haematoxylin.

### Evaluation and analysis of tumour-infiltrating immune cells

All images were captured using a BZ-X800 microscope (Keyence, Osaka, Japan) and the percentage of positive cells was measured visually. Three observers familiar with the pathological diagnoses performed the evaluations. Staining with anti-CD4, anti-CD8, anti-FoxP3, anti-CD20, anti-CD56, anti-CD68, anti-CD163, and anti-PD-1 monoclonal antibodies (mAbs) was evaluated by counting the number of positively stained cells in five different fields using a 10× eyepiece lens and a 40× objective lens, based on previous studies [16, 17]. Global evaluation of tumour infiltration was performed using the total number of cells. The number of positive cells was scored as follows and is shown in Table 1: + (≤30 cells/field), ++ (30–70 cells/field), +++ (70–120 cells/field), and ++++ (≥120 cells/field). Staining with anti-PD-L1 was evaluated by measuring the area of positive staining throughout the tissue, and areas with 1% or more were considered positive based on previous studies [18]. For PD-L1 staining counts, those positive in tumour cells were included, while those positive in immune cells, such as macrophages, were excluded. CD8 staining at the tumour cores and tumour margins were assessed by counting the number of positive cells in five different fields using a 10× eyepiece lens and a 40× objective lens. The cut-off values for high and low infiltration at the tumour cores were 100 cells/mm^2^, while those for high and low infiltration at the tumour margins were 200 cells/mm^2^, based on previous studies [19].

**Table 1:**
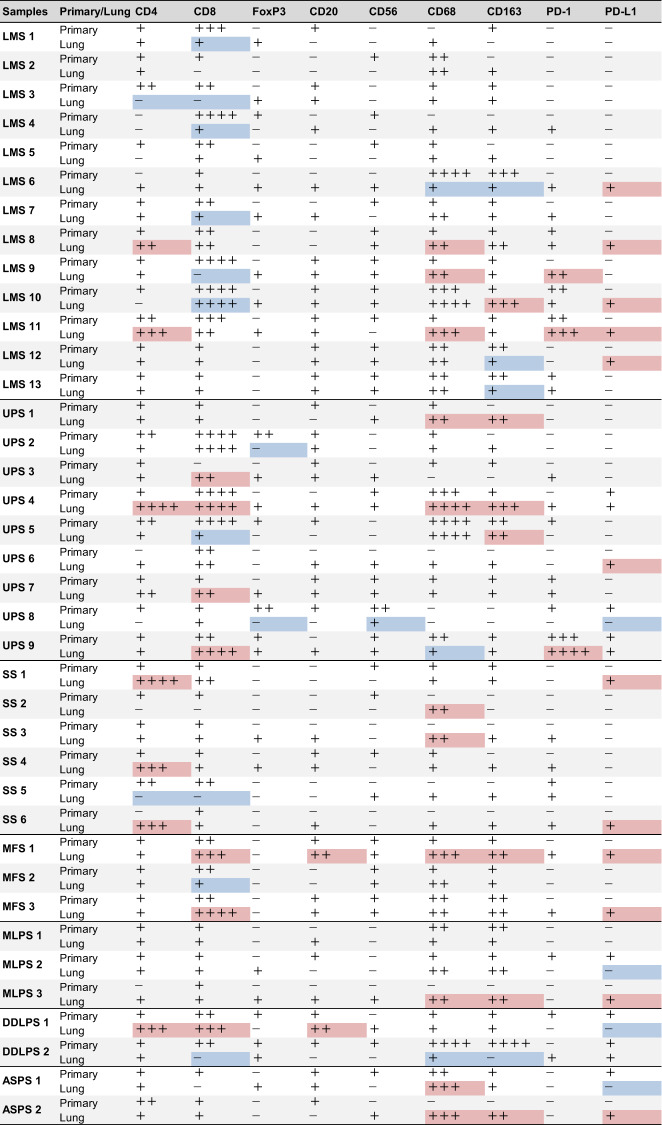
Analysis of immune cell infiltration using immunohistochemical staining of all soft tissue sarcoma samples.

Using a 40× objective lens, the number of positively stained cells in five different fields was counted for cluster of differentiation (CD)4, CD8, forkhead box protein 3 (FoxP3), CD20, CD56, CD68, CD163, and programmed cell death protein 1 (PD-1), and the total number was used to assess global tumour infiltration. The number of positive cells was scored as follows: + (≤30 cells/field), ++ (30–70 cells/field), +++ (70–120 cells/field), and ++++ (≥120 cells/field). Compared to the primary tumour, lung metastases with a marked increase were coloured red and those with a marked decrease were coloured blue. A marked increase or decrease was defined as an increase or decrease of more than 50% each, and those with a change of small absolute value, less than 30 cells were excluded. For PD-L1 staining counts, those positive in tumour cells were included, while those positive in immune cells, such as macrophages, were excluded. The area of PD-L1 positive staining against the entire tissue was measured and areas exceeding 1% were considered positive.

*LMS* leiomyosarcoma, *UPS* undifferentiated pleomorphic sarcoma, *SS* synovial sarcoma, *MFS* myxofibrosarcoma, *MLPS* myxoid liposarcoma, *DDLPS* dedifferentiated liposarcoma, *ASPS* alveolar soft part sarcoma.

### Statistical analysis

Evaluation of specimens stained with anti-CD4, anti-CD8, anti-FoxP3, anti-CD20, anti-CD56, anti-CD68, anti-CD163, and anti-PD-1 mAbs was performed using a Wilcoxon signed-rank test with the corresponding primary tumours and lung metastases, and the differences between the two groups were evaluated. Differences between two groups were considered statistically significant at *p* < 0.05. For the multivariate analysis of CD8, all 38 pairs of tumours included in this study were divided into two groups: tumours in which the number of CD8 positive cells decreased in lung metastasis compared to that in the primary tumour, and tumours in which the number remained the same or increased. The baseline characteristics of the two groups were compared. Univariate analysis was performed, followed by a multivariate logistic regression analysis. The multivariate regression model was performed using factors that showed marginal significance (*p* < 0.1) in the univariate analysis and those widely known to affect immunocompetence (age) [20]. Differences between the two groups were statistically analysed using the Mann–Whitney *U*-test for non-parametric continuous variables and Fisher’s exact test for categorical variables. Survival analyses were performed using publicly available data from The Cancer Genome Atlas (TCGA) cohort [21]. Using the CD8 T cell scores in the publicly available immune score data, the top 1/3 (26 patients) and bottom 1/3 (26 patients) of the 80 leiomyosarcoma patients were categorised into high and low CD8 score groups, respectively. A Cox proportional hazards model was used for multivariate survival analysis. For assessing overall survival and metastasis-free survival, multivariate analysis was performed by adding age, which generally correlates with prognosis, to CD8 score and tumour size, two factors that were significantly different in the univariate analysis. All statistical analyses were performed using JMP Pro version 16.0 (SAS Institute Inc., Cary, NC, USA).

### RNA extraction from formalin-fixed paraffin-embedded (FFPE) samples

All targeted tissue sections were classified as soft tissue LMS; uterine LMS was excluded. We targeted pairs of six primary foci and corresponding lung metastatic foci with a recent tissue collection time. From FFPE blocks, 4–24 unstained slides of 10 μm thickness were collected, and tumour sections were excised via macrodissection based on the corresponding haematoxylin and eosin (H&E) slides. Total RNA was isolated using the RNAstorm FFPE RNA Extraction Kit (Cell Data Sciences, Fremont, CA, USA), following the manufacturer’s instructions.

### Analysis of immune-related gene expression using the nCounter platform

We analysed the expression of 770 immune-related genes using the nCounter PanCancer Immune Profiling Panel (NanoString Technologies Inc., Seattle, WA, USA). Raw data were processed using nSolver Analysis Software (version 4.0; NanoString Technologies Inc.) for normalisation of mRNA expression, principal component analysis, hierarchical clustering, immune cell profiling, and functional pathway analysis. Pairwise differentially expressed genes (DEGs) were analysed using R software (The R Foundation for Statistical Computing, Vienna, Austria).

### Cell culture

The TYLMS-1 human LMS cell line was purchased from the Japanese Collection of Research Bioresources (JCRB) Cell Bank. TC616 is a human LMS cell line derived from the mediastinum and was created in our laboratory, as previously reported [22]. TYLMS-1 and TC616 cells were cultured at 37 °C under 5% CO_2_ in a 1:1 mixture of Dulbecco’s modified Eagle’s medium (DMEM) (Gibco) and Ham’s F-12 medium (Sigma-Aldrich, St. Louis, MO, USA) with 10% fetal bovine serum (FBS) (HyClone). Human CD8^+^ T cells were separated according to the manufacturer’s protocol using CD8 MicroBeads (Miltenyi Biotec, Bergisch Gladbach, Germany) after separating peripheral blood mononuclear cells (PBMC) from whole blood collected from healthy donors using Ficoll-Paque PLUS (Cytiva, Marlborough, MA, USA). CD8^+^ T cells were cultured at 37 °C under 5% CO_2_ in RPMI 1640 medium (Gibco) with 10% FBS and activated in advance for 48 h using anti-CD3 (BioLegend, San Diego, CA, USA), anti-CD28 (BioLegend), and human interleukin (IL)-2 (BioLegend) before being used for experiments.

### Inhibitor treatment

TYLMS-1 and TC616 cells were treated to inhibit epithelial cellular adhesion molecule (EPCAM) signalling with 40 μM tumour necrosis factor-α converting enzyme (TACE) inhibitor (TAPI)-1 (Selleck Chemicals, Houston, TX, USA), 10 μM γ-secretase inhibitor (DAPT) (Selleck Chemicals), or a combination of both inhibitors when the cells reached ~60% confluence. Drug type and concentration were determined based on previous studies [23]. The inhibitors were administered once at 0 h in all the cultures. For the Transwell assay, cells were cultured in RPMI 1640 medium supplemented with 0.5% FBS at the time of inhibitor administration and incubated for 48 h to produce conditioned media (CM).

### Creation of knockdown cells

TYLMS-1 and TC616 cell suspensions (in 10% FBS-containing DMEM: Ham’s F-12 medium without antibiotics) were seeded and cultured in 12-well plates. In both TYLMS-1 and TC616 cells, small interfering (si)RNA was transfected when the cells reached ~60% confluence. For EPCAM knockdown, a Silencer Select Pre-Designed siRNA (s529200; s529201, Thermo Fisher Scientific, Waltham, MA, USA) was transfected using Lipofectamine 3000 (Thermo Fisher Scientific), following the manufacturer’s protocol. For double knockdown of EPCAM and synaptosomal-associated protein of 25 kDa (SNAP25), Silencer Select Pre-Designed siRNA (s529200, Thermo Fisher Scientific) and Silencer Select Pre-Designed siRNA (s13189, Thermo Fisher Scientific) were simultaneously transfected into TYLMS-1 cells. Control cells were transfected with Silencer Negative Control No.1 siRNA (AM4611, Thermo Fisher Scientific). The cells were used for experiments 48 h after transfection. When used in the Transwell assay, the medium was replaced with 0.5% FBS-supplemented RPMI 1640 medium 48 h after transfection, and the cells were further cultured for 48 h to produce CM.

### Quantitative reverse transcription-polymerase chain reaction (RT-qPCR)

RNA was isolated from cells using the RNeasy Mini Kit (Qiagen, Hilden, Germany), and reverse transcription was performed using the PrimeScript RT Reagent Kit (Takara Bio, Shiga, Japan). RT-qPCR was performed using TB Green Premix Ex Taq II (Takara Bio). The reactions were performed using a CFX Connect Real-Time PCR Detection System (Bio-Rad, Hercules, CA, USA). Primer sequences for the detection of EPCAM were 5′-TGCTGGAATTGTTGTGCTGG-3′ and 5′- AAGATGTCTTCGTCCCACGC-3′ and for SNAP25 were 5′-CGTCGTATGCTGCAACTGGTTG-3′ and 5′-GGTTCATGCCTTCTTCGACACG-3′. All samples were normalised to GAPDH using the 5′-AATTCCATGGCACCGTCAAG-3′ and 5′-ATCGCCCCACTTGATTTTGG-3′ primers.

### Western blotting

For western blotting, tumour cells were washed and lysed with CellLytic M (Sigma-Aldrich) containing protease and phosphatase inhibitors. The lysate was cleared by centrifugation at 15,000 rpm for 15 min and denatured at 95 °C for 5 min. The lysate was stored at −80 °C until immunoblot analysis. Proteins were separated by electrophoresis using a NuPAGE 4–12% Bis-Tris Gel (Thermo Fisher Scientific) and transferred to Amersham Protran (Cytiva). After blocking the membrane with 5% skim milk for 1 h, it was incubated overnight at 4 °C with specific primary antibodies. After hybridisation with secondary antibodies, the protein bands were visualised using Amersham ECL western blotting detection reagent (Cytiva). The antibodies used in this study are listed as follows; anti-EPCAM (#ab223582, Abcam), anti-SNAP25 (#5308, Cell Signaling Technology), anti-Actin (#MA5-11869, Invitrogen), anti-rabbit IgG (#7074, Cell Signaling Technology), and anti-mouse IgG (#7076, Cell Signaling Technology).

### Transwell assay

The prepared CM was centrifuged at 3500 rpm for 30 min to remove the cell debris. CM (500 μl) was added to the lower chamber of a 6.5 mm Transwell with a 5.0 μm Pore Polycarbonate Membrane Insert (Corning, New York, NYC, USA), and CD8^+^ T cells (5 × 10^5^ cells/100 μl) resuspended in RPMI 1640 medium were added to the upper chamber. After incubation for 6 h at 37 °C under 5% CO_2_, cells that had migrated to the lower side of the membrane were collected, fixed with Diff-Quik Fix, stained with Diff-Quik II, and observed. All images were captured using a BZ-X800 microscope (Keyence). The number of migrated cells was measured visually in five random fields at ×100 magnification, and the sum was scored for evaluation.

### RNA sequencing and data analysis

We created three clones of TYLMS-1 cells treated with TAPI + DAPT, three clones of untreated TYLMS-1 cells (dimethyl sulfoxide added), three clones of TYLMS-1 cells transfected with siEPCAM, and three clones of TYLMS-1 cells transfected with scrambled siRNA, for a total of 12 TYLMS-1 cell clones. Total RNA was extracted from the clones using a RNeasy Mini Kit (Qiagen). RNA quality was assessed using an Agilent 2100 Bioanalyzer (Agilent Technologies, Santa Clara, CA, USA). RNA sequencing (RNA-seq) libraries were prepared using the Illumina Stranded-specific library preparation method (dUTP method) and sequenced as paired-end 150 base pair (bp) reads on a NovaSeq 6000 machine. Raw RNA-Seq data were subjected to FastQC for quality control. The data were processed using BioJupies [24] for the normalisation of mRNA expression and DEG analysis.

### Proliferation assay

TYLMS-1 and TC616 cells were seeded in 96-well plates at 4.0 × 10^3^ cells and 2.0 × 10^3^ cells per well, respectively. Four hours after seeding, the cell viability in each well was measured as a standard value using the CellTiter-Glo 2.0 Cell Viability kit (Promega, Madison, WI, USA). The relative cell viability was monitored every 24 h for up to 72 h. The assay was read on an EnSight Multimode Plate Reader (PerkinElmer, Waltham, MA, USA).

### Migration assay

Tumour cell migration was assessed using a 6.5 mm Transwell with an 8.0 µm Pore Polycarbonate Membrane Insert (Corning). Knockdown-treated TYLMS-1 cells (5.0 × 10^4^/200 µl) or TC616 cells (2.0 × 10^4^/200 µl) were added to the upper chamber and 600 µl of medium with 10% FBS to the lower chamber. After 16 h of incubation at 37 °C under 5% CO_2_, migrated cells were fixed with Diff-Quik Fix, stained with Diff-Quik II, and observed. All images were captured using a BZ-X800 microscope (Keyence). The number of migrated cells was visually measured in five random fields at ×100 magnification, and the sum was scored for evaluation.

## Results

### Comparison of immune profiles between primary tumours and lung metastases in STS

**Fig. 1:**
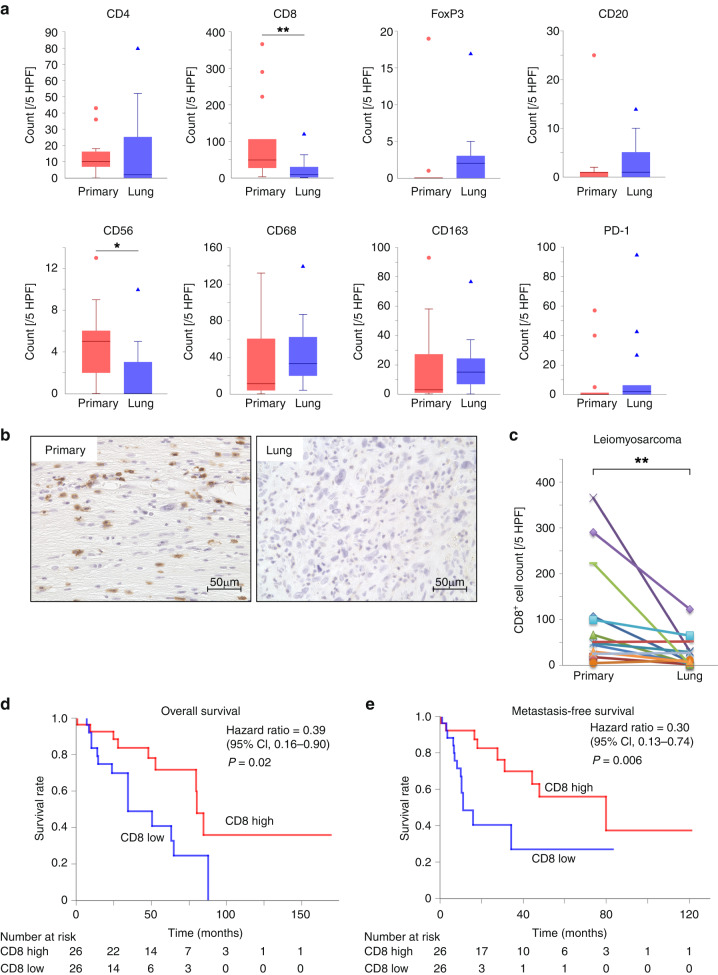
Decreased tumour-infiltrating cluster of differentiation (CD)8^+^ cell numbers in lung metastasis of leiomyosarcoma and correlation between the abundance of infiltrating CD8^+^ T cells and prognosis in leiomyosarcoma. **a** Comparison of tumour-infiltrating immune cell numbers in primary and lung metastatic leiomyosarcoma tissues (*n* = 13). **b** Representative images of CD8 immunohistochemical (IHC) staining of leiomyosarcoma. These were primary and lung metastatic tissues from the same patient. **c** Comparison of CD8^+^ cell numbers in primary and lung metastatic leiomyosarcoma tissues (*n* = 13). The lines indicate that they were obtained from the same patient. **d**, **e** The data were obtained from The Cancer Genome Atlas cohort. Of the patients with 80 leiomyosarcomas, the top (26 patients) and bottom 1/3 (26 patients) were categorised into the high and low CD8 score groups, respectively. **d** Comparison of the overall survival of patients with leiomyosarcoma between the high and low CD8 score groups. **e** Comparison of metastasis-free survival of patients with leiomyosarcoma between high and low CD8 score groups. **a**, **c** Wilcoxon signed-rank test. *p < 0.05, **p < 0.01. **d**, **e** Log-rank test.

Immune cell infiltration profiles were created via immunostaining using paired tissue samples from the primary and lung metastatic sites of 38 STS samples. The histological types and clinical information are shown in Supplementary Table 1, and the immune cell counts are shown in Table 1. We performed a paired analysis to compare the changes between the primary tumour and lung metastasis. In the analysis of all cases, CD68, CD163 and PD-1 were significantly increased in lung metastases (*p* = 0.01, *p* = 0.04 and *p* = 0.04, respectively, Supplementary Fig. 1a). Next, we performed the same analysis on three tissue types: LMS (*n* = 13), undifferentiated pleomorphic sarcoma (*n* = 9), and synovial sarcoma (*n* = 6), all of which had relatively large sample sizes. Significant differences were observed in CD8 and CD56 expression in LMS, and CD68 expression in synovial sarcoma (*p* = 0.003, *p* = 0.03 and *p* = 0.03, respectively, Fig. 1a and Supplementary Fig. 1b, c). Notably, CD8 expression in LMS was reduced by ~1/4 in lung metastases.

### In lung metastases of LMS, CD8^+^ T cells are significantly reduced compared to primary tumours

We focused on CD8^+^ cells in the LMS and conducted further analyses. Representative examples of immunostaining are shown in Fig. 1b. We performed a paired analysis of primary tumours and lung metastases in LMS and other sarcomas (Fig. 1c and Supplementary Fig. 2). The results showed no significant changes in CD8^+^ cells in lung metastases of other sarcomas, and the significant decrease in CD8^+^ cells in lung metastases only in LMS. In contrast, patient background factors, such as sex, age, chemotherapy, and radiotherapy are known to contribute to changes in infiltrating immune cells [12, 21, 25, 26]. In addition, in STS, tumour-infiltrating lymphocytes are more abundant in non-translocation-associated sarcomas, including LMS, than in translocation-associated sarcomas [27]. Therefore, we conducted multivariate analysis to ensure that these factors were not involved (Supplementary Table 2a). The results showed that LMS was the only factor involved in decreasing the number of CD8^+^ cells in the lung metastases (*p* = 0.04). The infiltration of CD8^+^ T cells into tumours is related to the prognosis of numerous malignant tumours; in most cases, low infiltration is associated with poor prognosis [28, 29]. We analysed the correlation between CD8^+^ T cell scores and prognosis in LMS using TCGA database. The low CD8^+^ T cell score group had a significantly worse prognosis in terms of overall survival and metastasis-free survival (Fig. 1d, e). The results of the additional multivariate analyses also supported these results (Supplementary Table 2b, c). Therefore, low infiltration of CD8^+^ T cells in lung metastases of LMS is considered a poor prognostic factor. We therefore decided to further analyse this mechanism.

### CD8^+^ T cell infiltration in lung metastases of LMS is reduced at the tumour cores rather than at the tumour margins, resulting in heterogeneous distribution

We first analysed changes in the spatial distribution of infiltrating CD8^+^ T cells in the LMS. For the infiltration of CD8^+^ T cells into the tumour core and tumour margins, we classified four groups according to the number of CD8^+^ T cells: tumour core high/tumour margin high, tumour core high/tumour margin low, tumour core low/tumour margin high and tumour core low/tumour margin low, based on previous studies [19]. Primary tumours had 4, 3, 2, and 4 cases, respectively, and tumour cores were high in more than half of the cases (Supplementary Fig. 3a). In contrast, lung metastases accounted for 1, 2, 3, and 7 cases, respectively, with tumour cores low in approximately three-quarters of the cases (Supplementary Fig. 3b). Quantitative comparison showed that the number of infiltrating CD8^+^ T cells in the tumour cores in lung metastases was significantly lower than in tumour margins (*p* = 0.01, Supplementary Fig. 3c, right). In contrast, the number of infiltrating CD8^+^ T cells in the tumour cores in primary tumours did not differ from that in tumour margins (Supplementary Fig. 3c, left). The margin/core ratio of the number of infiltrating CD8^+^ T cells also showed that there were more infiltrating CD8^+^ T cells at the tumour margins in lung metastases (*p* = 0.03, Supplementary Fig. 3d), indicating heterogeneity in the distribution of CD8^+^ T cells between the tumour core and margin. Therefore, some immune evasion mechanisms that inhibit the infiltration of CD8^+^ T cells into the tumour core were considered to be present in lung metastases of LMS.

### Reduced CD8^+^ T cell infiltration in lung metastases of LMS can be confirmed at the gene expression level, with decreased T cell function and chemokine-related pathways

**Fig. 2:**
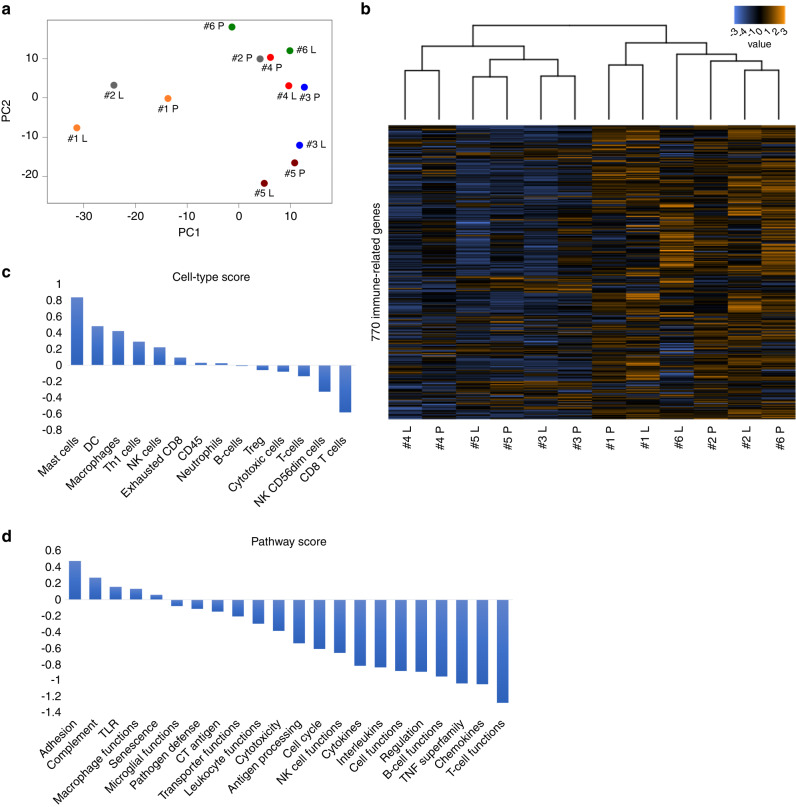
Analysis of gene expression profiles determined by the nCounter® PanCancer Immune Profiling Panel in primary and lung metastases of leiomyosarcoma in six patients. **a** Principal component analysis. The same numbers indicate the same patient, with P indicating primary tumour and L indicating lung metastasis. **b** Correlation heatmap of unsupervised hierarchical clustering. The same numbers indicate the same patient. **c** Trend plot of tumour-infiltrating immune cell scores calculated using nSolver 4.0. **d** Trend plots of pathway scores calculated using nSolver 4.0.

Known factors that induce the exclusion of CD8^+^ T cells include sustained high PD-L1 expression and the loss of HLA class I [30, 31]. We first analysed the correlation between the expression of these molecules and the number of infiltrating CD8^+^ T cells. The number of infiltrating CD8^+^ T cells tended to be higher in the PD-L1 positive group than in the PD-L1 negative group; however, this difference was not statistically significant (*p* = 0.07, Supplementary Fig. 4a, b). This result did not mean that sustained high PD-L1 expression excluded CD8^+^ T cells [32]. There was no difference in the number of infiltrating CD8^+^ T cells between the high- and low-HLA class I expression groups (Supplementary Fig. 4c, d). Because no known mechanisms are likely to be involved, we hypothesised that novel mechanisms may be involved. Therefore, we performed gene expression analysis to analyse the variation in gene expression involved in immunity. We performed gene analysis using nCounter on tissue specimens from the primary and lung metastatic sites of the six most recently diagnosed patients with LMS among our 13 patients. Principal component analysis showed that in five patients, excluding patient no. 2, the primary tumours and lung metastases in the same case were in close proximity (Fig. 2a). Similarly, hierarchical clustering analysis tended to cluster the primary tumours and lung metastases in the same case rather than by primary tumours or lung metastases (Fig. 2b). This showed that in our LMS patients, the expression profiles of immune-related genes were roughly similar between the primary tumours and lung metastases in the same cases. Furthermore, in the cell type score, CD8^+^ T and natural killer (NK) cells were reduced in lung metastases, which matched our immunostaining results (Figs. 1a and 2c). Regarding the pathway score, we observed a decrease in T cell function and chemokine pathways in lung metastases (Fig. 2d). In summary, the reduction in infiltrating CD8^+^ T cells in lung metastases of LMS confirmed by immunohistochemical analysis was also confirmed at the gene expression level, and furthermore, the pathways associated with immune cell migration were reduced.

### EPCAM was upregulated in lung metastases of LMS and correlated with the number of infiltrating CD8^+^ T cells

**Fig. 3:**
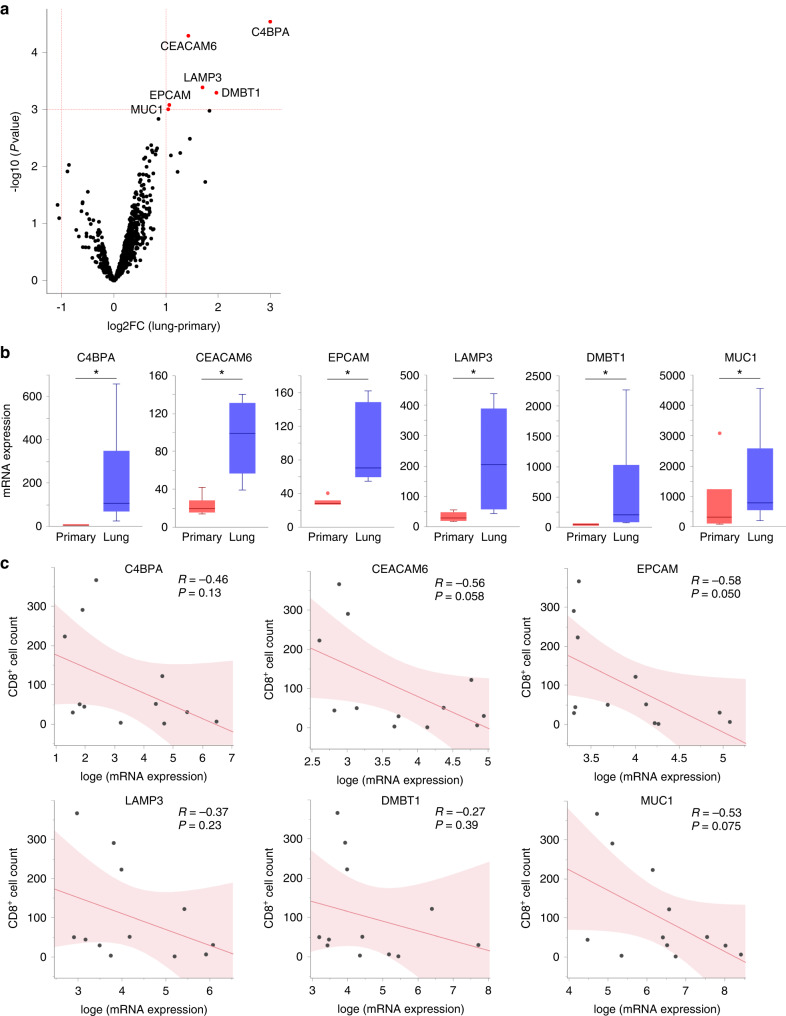
Analysis of differentially expressed genes in primary and lung metastases of leiomyosarcoma in six patients. Furthermore, correlation analysis was performed between the expression of the identified genes and the number of tumour-infiltrating CD8^+^ cells. **a** Volcano plot showing differentially expressed genes (DEGs) in corresponding lung metastases compared to primary leiomyosarcoma. Red dots indicate genes with statistically significant differences (fold-change >1.0, *p* < 0.001). **b** Comparison of gene expression levels in primary and lung metastases for each DEG. **c** Correlation analysis between gene expression levels and the number of tumour-infiltrating CD8^+^ cells based on immunohistochemical staining for each DEG (*n* = 12; 6 patients with primary tumours and 6 with lung metastasis). Gene expression levels were converted to natural logarithms. R represents the Pearson’s correlation coefficient. **a**, **b** DEG analysis was performed using the R package limma.

Next, to identify the genes involved in the exclusion of CD8^+^ T cells from lung metastases of LMS, we analysed the DEGs and identified six genes: *C4BPA*, *CEACAM6*, *EPCAM*, *LAMP3*, *DMBT1*, and *MUC1* (Fig. 3a, b). When we analysed the correlation between the expression levels of these genes and the number of CD8^+^ T cells, we found the strongest negative correlation for *EPCAM* (Fig. 3c). We also examined the expression of each gene in normal body tissues and found that all five genes, except for *EPCAM*, were highly expressed in the respiratory system (The Human Protein Atlas; data not shown). Therefore, DEGs other than *EPCAM* may have been contaminated with normal lung tissues. Based on these results, we hypothesised that the upregulation of *EPCAM* in lung metastases from LMS may be involved in the exclusion of CD8^+^ T cells. This effect was verified in vitro.

### In human LMS cell lines, inhibition or knockdown of EPCAM restored the migration of CD8^+^ T cells

**Fig. 4:**
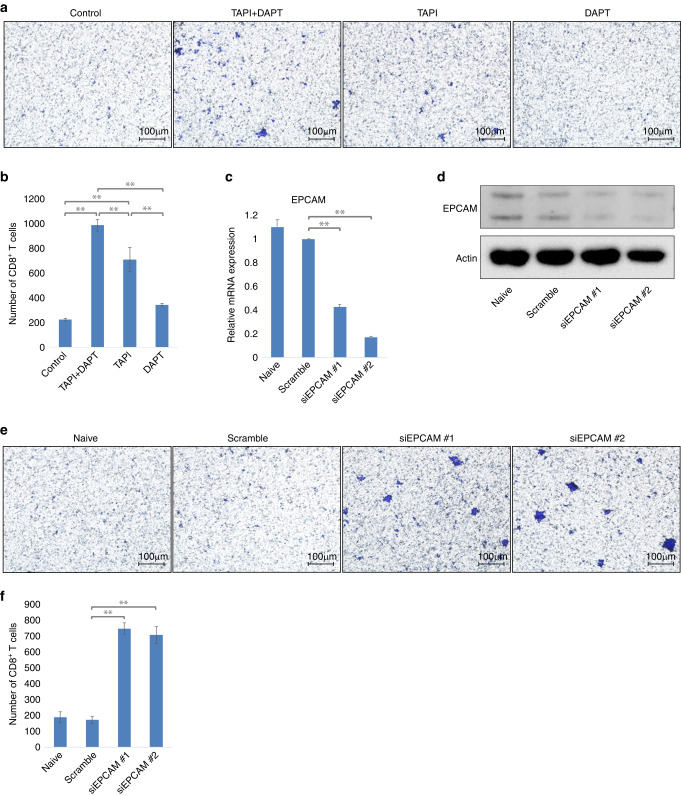
Analysis of the effect of epithelial cellular adhesion molecule (EPCAM) inhibition and knockdown on the migration of cluster of differentiation (CD)8^+^ T cells in the human leiomyosarcoma cell line TYLMS-1. **a**, **b** TYLMS-1 cells treated with EPCAM inhibitors-produced conditioned media. Tumour necrosis factor-α converting enzyme (TACE) inhibitor (TAPI), and γ-secretase inhibitor (DAPT) were used. In addition to the inhibition of EPCAM signalling by the combination of TAPI and DAPT, partial inhibition of EPCAM signalling was performed with each drug alone. The migration of CD8^+^ T cells was evaluated and compared using a Transwell assay with conditioned medium. **c**–**f** TYLMS-1 cells were transfected with small interfering (si)EPCAM or scrambled siRNA-produced conditioned medium. The migration of CD8^+^ T cells was evaluated and compared using a Transwell assay with conditioned medium. **a**, **e** The membrane of the Transwell insert was stained, and the number of migrated CD8^+^ T cells was evaluated. Representative microscopy images are shown. **b**, **f** Comparison of the numbers of migrated CD8^+^ T cells. For each membrane shown in (**a**) and (**e**), the number of stained positive cells was counted in five different fields using a 10× objective lens, and the total number was evaluated. **c** EPCAM expression in each cell line was determined by reverse transcription-quantitative polymerase chain reaction (RT-qPCR). **d** EPCAM expression in each cell line was determined by western blotting. Data in (**b**) and (**f**) are mean ± standard deviation (SD); *n* = 3. Two-tailed *t*-test. *p < 0.05, **p < 0.01.

We performed a Transwell assay using the human LMS cell line TYLMS-1 and CD8^+^ T cells isolated from healthy donors (Supplementary Fig. 4e). First, we performed an experiment inhibited EPCAM signalling. Through two-step proteolytic processing, EPCAM is sequentially cleaved by TACE and presenilin 2 (PS-2), a protease component of γ-secretase complex, and releases an N-terminal extracellular domain (EpEX) and a 5 kDa C-terminal intracellular domain (EpICD) to initiate signal transduction [33]. To inhibit EPCAM signalling, both TACE inhibitor (TAPI), which block the release of EpEX, and γ-secretase inhibitor (DAPT), which, in turn, block the release of EpICD, are used [23, 34]. Significantly more migrated CD8^+^ T cells were observed in the TAPI + DAPT and TAPI-only groups (Fig. 4a, b). Next, we conducted EPCAM knockdown via siRNA transfection (Fig. 4c, d) and performed the same Transwell assay. In TYLMS-1 cells with EPCAM knockdown, the number of migrated CD8^+^ T cells significantly increased (Fig. 4e, f). In addition, when we performed the same experiment using another LMS cell line, TC616, we observed a significant increase in the number of migrated CD8^+^ T cells following EPCAM inhibition by TAPI + DAPT and EPCAM knockdown by siEPCAM transfection (Supplementary Fig. 5a–f). In summary, when EPCAM was inhibited or knocked down in human LMS cell lines, CD8^+^ T cell migration increased. Thus, the upregulation of EPCAM in human LMS cell lines inhibited the migration of CD8^+^ T cells.

### The restoration of CD8^+^ T cell migration by knockdown of EPCAM in human LMS cell lines is cancelled by simultaneous knockdown of SNAP25

**Fig. 5:**
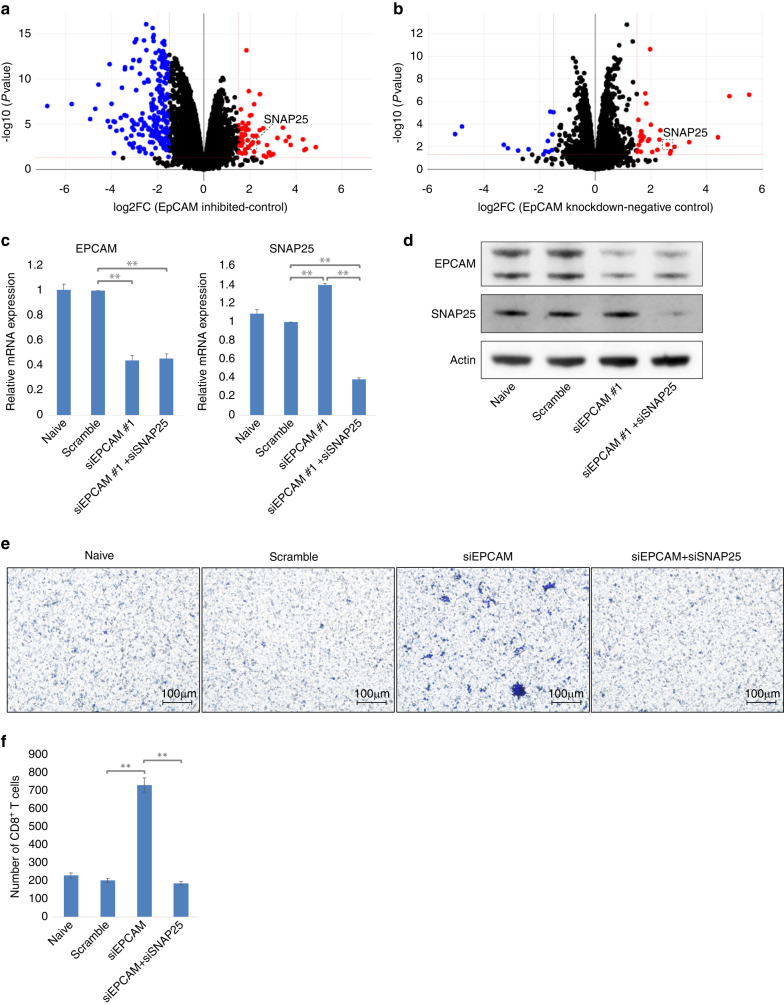
Analysis of differentially expressed genes and the effect of synaptosomal-associated protein of 25 kDa (SNAP25) knockdown on cluster of differentiation (CD)8^+^ T cell migration in epithelial cell adhesion molecule (EPCAM)-inhibited and knockdown human leiomyosarcoma cell line TYLMS-1. **a** Volcano plot showing differentially expressed genes in TYLMS-1 treated with Tumour necrosis factor-α-converting enzyme (TACE) inhibitor (TAPI) + γ-secretase inhibitor (DAPT) compared to control TYLMS-1 (dimethyl sulfoxide added). Red and blue dots indicate genes with statistically significant differences (fold-change >1.5, *p* < 0.05). **b** Volcano plot showing differentially expressed genes in TYLMS-1 with small interfering (si)EPCAM transfection compared to TYLMS-1 with scramble siRNA transfection. Red and blue dots indicate genes with statistically significant differences (fold-change >1.5, *p* < 0.05). **c**–**f** Conditioned media were prepared by transfection of siEPCAM + siSNAP25, siEPCAM, or scramble siRNA into TYLMS-1 cells. The migration of CD8^+^ T cells was evaluated and compared using a Transwell assay with conditioned medium. **c** The expression of EPCAM and SNAP25 in each cell line was determined using reverse transcription-quantitative polymerase chain reaction (RT-qPCR). **d** Expression of EPCAM and SNAP25 in each cell line was determined using western blotting. **e** The membrane of the Transwell insert was stained, and the number of migrated CD8^+^ T cells was evaluated. Representative microscopic images are presented. **f** Comparison of the number of migrated CD8^+^ T cells. In each membrane shown in (**e**), the number of stained positive cells was counted in five different fields using a 10× objective lens, and the total number was evaluated. Data are presented as the mean ± standard deviation (SD), *n* = 3. Two-trailed *t*-test. *p < 0.05, **p < 0.01.

To our knowledge, no previous studies have examined the relationship between EPCAM and CD8^+^ T cell migration. First, we examined the chemokines involved in CD8^+^ T cell migration [35, 36]. However, when we examined whether the expression levels of C-C motif chemokine ligand (CCL5), C-X-C motif chemokine ligand (CXCL)9, CXCL10, CXCL11, and CX3CL1 were altered by the inhibition of signal transduction or EPCAM knockdown in human LMS cell lines, we observed no consistent changes in any of the cytokines (data not shown). Therefore, we performed mRNA-seq to comprehensively analyse this mechanism. In the analysis of DEGs, 65 upregulated and 208 downregulated genes were identified in the EPCAM-inhibited TYLMS-1 cells compared to those in the control (Fig. 5a). Compared to the control, 29 upregulated and 14 downregulated genes were identified in the EPCAM-knockdown TYLMS-1 cells (Fig. 5b). In the search for common genes in each set of DEGs, only *SNAP25* upregulation was confirmed (Supplementary Fig. 6a). Next, we investigated whether SNAP25 mediated the inhibition of CD8^+^ T cell migration by EPCAM in TYLMS-1 cells. As there were no specific inhibitors of SNAP25, we determined whether double knockdown of EPCAM and SNAP25 could prevent the increase in migrated CD8^+^ T cells observed with a single knockdown of EPCAM (Fig. 5c, d). With double knockdown, the number of migrated CD8^+^ T cells decreased to the same level as in the naïve and control groups (Fig. 5e, f). In addition, when we performed the same experiment using another LMS cell line, TC616, we observed a decrease in the number of migrated CD8^+^ T cells with double knockdown to the same level as in the naïve and control groups (Supplementary Fig. 6b–e). In summary, in human LMS cell lines with knockdown and inhibition of EPCAM, the expression of *SNAP25* was significantly increased. SNAP25 and EPCAM knockdown in vitro abrogated the increase in CD8^+^ T cell migration observed after EPCAM knockdown. This suggests that SNAP25 mediates the effect of EPCAM on CD8^+^ T cell migration in the human LMS cell lines.

### Upregulation of EPCAM in human LMS cell lines does not affect cell proliferation but promotes cell migration

Finally, EPCAM is reportedly involved in cell proliferation and migration [23, 33, 34]. Therefore, we investigated the effect of EPCAM expression on cell proliferation and migration in LMS cell lines. In both TYLMS-1 and TC616 cells, EPCAM-knockdown cells showed no differential proliferation compared to control cells (even in double knockdown cells with EPCAM and SNAP25) (Supplementary Fig. 7a, b). In contrast, in both TYLMS-1 and TC616 cells, EPCAM-knockdown cells showed significantly reduced cell migration compared with control cells (cell migration was equally reduced in double knockdown cells with EPCAM and SNAP25) (Supplementary Fig. 7c–f). These results indicate that upregulation of EPCAM in LMS does not affect tumour growth but may promote metastasis. Therefore, it is considered that EPCAM upregulation in LMS promotes metastasis and inhibits CD8^+^ T cell infiltration, thus worsening the prognosis by a dual mechanism.

## Discussion

In this study, we analysed the differences in the immune environment between primary and lung metastatic lesions of STS using clinical samples and found that the infiltration of CD8^+^ T cells into the tumour core was significantly reduced in lung metastases of LMS. Gene expression analysis suggested that *EPCAM* might be responsible for decreased CD8^+^ T cell infiltration in lung metastases. In vitro experiments using human LMS cell lines further demonstrated that EPCAM inhibited the migration of CD8^+^ T cells via SNAP25. This study is the first to reveal a novel and unique role for EPCAM, suggesting that it may contribute to immune evasion by inhibiting the infiltration of CD8^+^ T cells in lung metastases of LMS.

In recent years, immunotherapy has shown good outcomes for some malignant tumours [5]. Among them, immune checkpoint inhibitors have shown good therapeutic outcomes against solid tumours, such as melanoma, non-small cell lung cancer, renal cell carcinoma, and head and neck cancer [6–9]; however, the cancer types are limited, and numerous malignant tumours are not responsive [10]. Several studies have reported limited therapeutic effects of immune checkpoint inhibitors in STS [37]. In a phase 2 clinical trial (SARC028) evaluating the therapeutic effect of pembrolizumab for advanced STS, therapeutic effects were observed in some undifferentiated pleomorphic sarcomas and liposarcomas; however, the effect was limited, and no therapeutic effect was reported in LMS [11]. In our study, CD8^+^ T cell infiltration was significantly reduced in LMS lung metastases. The therapeutic effects of immune checkpoint inhibitors correlate with the abundance of infiltrating CD8^+^ T cells [38]. Although this study only examined lung metastases, the lack of infiltrating CD8^+^ T cells may explain the lack of a therapeutic effect of pembrolizumab in LMS in SARC028. Furthermore, regardless of immunotherapy, the abundance of tumour-infiltrating CD8^+^ T cells correlated with good prognosis in numerous malignant tumours [28, 29]. The role of tumour-infiltrating CD8^+^ T cells in LMS is not clear; therefore, when we analysed it using cohort data, we found that the abundance of tumour-infiltrating CD8^+^ T cells correlated with prognosis. Therefore, an approach to recover tumour-infiltrating CD8^+^ T cells, which are reduced in distant metastases, can improve the prognosis of LMS.

Cytokines play a significant role in the recruitment of CD8^+^ T cells to tumours. CCL5, CXCL9, CXCL10, CXCL11, and CX3CL1 are involved in the recruitment of CD8^+^ T cells to tumours [35, 36]. Gene expression analysis revealed no differences in the expression of these cytokines between LMS primary lesions, which had a relatively high number of infiltrating CD8^+^ T cells, and LMS lung metastases, which had a lower number of infiltrating CD8^+^ T cells. Thus, we focused on *EPCAM* by considering the gene expression results. In vitro verification revealed that EPCAM expression in human LMS cell lines reduced CD8^+^ T cell migration. EPCAM is a type I transmembrane glycoprotein that functions homophilically as an epithelium-specific intercellular adhesion molecule. Additionally, EPCAM plays a role in signal transduction, cell migration, proliferation, and differentiation [39]. EPCAM is highly expressed in numerous malignant tumours, and much research has been conducted on its use as a prognostic marker [40]. It is also used as a cancer-associated antigen and has been used in clinical practice as an EPCAM/CD3 bispecific antibody formulation for refractory malignant ascites [41]. It has also been investigated as a therapeutic target, and clinical trials of anti-EPCAM monoclonal antibodies have been conducted in colorectal cancer and metastatic breast cancer [42, 43]. Although these drugs are well tolerated, their therapeutic efficacy is limited, and they are not currently used. However, EPCAM is still considered a promising therapeutic target and is being investigated as a novel antibody preparation and target antigen for CAR-T therapy [44]. There are few reports on the role of EPCAM in tumour immunity. Moreover, there are only reports of its involvement in PD-L1 protein expression in colorectal cancer and the cytotoxic activity of NK cells in hepatocellular carcinoma [34, 45]. This study found that EPCAM can also be used as a target for immunotherapy.

To explore the mechanism by which EPCAM inhibits the migration of CD8^+^ T cells, we conducted RNA-seq in EPCAM-knockdown and EPCAM-inhibited human LMS cell lines and performed a comprehensive analysis. The data revealed that the expression of *SNAP25* significantly increased with EPCAM knockdown and inhibition. When SNAP25 was knocked down with EPCAM in vitro, an increase in the number of migrating CD8^+^ T cells was abrogated. Therefore, SNAP25 is considered to mediate the inhibition of CD8^+^ T cell migration by EPCAM. SNAP25 is one of the proteins that make up the SNARE complex located in the presynaptic membrane and is widely known as a protein essential for the exocytosis of neurotransmitters. SNAP25 has long been associated with attention deficit hyperactivity disorder and schizophrenia [46]; while there have been almost no reports related to malignant tumours. However, a recent study using TCGA database reported that SNAP25 expression correlates with tumour infiltration of immune cells in colon and prostate cancers [47, 48]. Di et al. reported that the expression of SNAP25 in prostate cancer was positively correlated with the infiltration of immune cells such as B, CD8^+^ T, CD4^+^ T, neutrophils, dendritic cells, macrophages, and NK cells [48]. In addition, the expression of SNAP25 was positively correlated with chemokine/chemokine receptors, suggesting that SNAP25 may regulate the migration of immune cells. In this study, we found that SNAP25 is involved in the migration of CD8^+^ T cells in human LMS cell lines. However, further research is required to elucidate the detailed mechanism by which SNAP25 is involved in immune cell migration.

The limitations of this study include its small sample size. We analysed 13 samples of LMS by immunostaining and six by gene expression analysis and identified EPCAM as a gene involved in the decrease in infiltrating CD8^+^ T cells in lung metastases of LMS. Nonetheless, the sample size was insufficient, and more samples should be examined in the future. In addition, we only studied primary and lung metastatic lesions and did not examine metastases to other organs, such as the bone, liver, brain, and intestine. Although the lungs are the most common organ for distant metastases of LMS, what happens in cases of metastasis to other organs remains unknown. Next, we showed in vitro that the knockdown or inhibition of EPCAM in human LMS cell lines increased the migration of CD8^+^ T cells; however, this needs to be verified in vivo. As no cell lines or animal models are readily available for LMS, patient-derived cell or xenograft models must be used for in vivo verification. In addition, because the verification of these findings requires the evaluation of tumour infiltration by CD8^+^ T cells, animal models with functional immune cells, such as humanised mice, are required.

In this study, the number of infiltrating CD8^+^ T cells was significantly reduced in lung metastases of LMS compared with that in primary lesions. Furthermore, gene expression data showed that EPCAM is upregulated in lung metastases, suggesting that it is involved in the decrease in infiltrating CD8^+^ T cells, which was verified in vitro. To our knowledge, this is the first study to report a unique role of EPCAM in the suppression of CD8^+^ T cell migration. In addition, EPCAM knockdown in LMS cell lines reduced tumour cell migration in vitro. In LMS, therapies targeting EPCAM could potentially improve prognosis by reducing metastases and restoring the infiltration of CD8^+^ T cells.

### Supplementary information

Supplementary Data Legends

Supplementary Table 1

Supplementary Table 2a, 2b, 2c

Supplementary Figure 1

Supplementary Figure 2

Supplementary Figure 3

Supplementary Figure 4

Supplementary Figure 5

Supplementary Figure 6

Supplementary Figure 7

## Data Availability

The data used in this study are available from the corresponding author upon reasonable request. In the handling of human specimens in this study, it has been stipulated not to publish gene expression data, which has been approved by the IRB of Kyushu University. We therefore decided not to publish gene expression data of the patient-derived leiomyosarcoma used in this study. The gene expression data of human leiomyosarcoma cell line TYLMS-1 are available in the Gene Expression Omnibus (GEO) repository with the accession number GSE250089.
