# Supplementary material for: Immune evasion in lung metastasis of leiomyosarcoma: upregulation of EPCAM inhibits CD8+ T cell infiltration
Source: Br J Cancer. 2024 Jan 30;130(7):1083–95. doi: 10.1038/s41416-024-02576-z (PMC10991329; doi:10.1038/s41416-024-02576-z)
Supplement: Supplementary file 1 — Supplementary Data Legends [file 41416_2024_2576_MOESM1_ESM.docx]

**Supplementary Data Legends**

Supplementary Table 1. Histological types and clinical features of the target samples in this study.

Supplementary Table 2.

a. Multivariate analysis of factors involved in the decrease of the tumour-infiltrating cluster of differentiation (CD)8^+^ cell numbers in lung metastasis. The 38 soft tissue sarcomas included in this study were divided into two groups: tumours in which the number of CD8^+^ cells decreased in lung metastases compared to that in the primary tumour and tumours in which the number remained the same or increased (*n* = 19 and *n* = 19, respectively). The baseline characteristics of the two groups were compared, and a multivariate logistic regression model was constructed based on the results of the univariate analysis. For non-parametric continuous variables, the Mann–Whitney U test was performed. For categorical variables, Fisher's exact test was performed.

b, c. A Cox proportional hazards model was used for univariate and multivariate survival analyses of 80 patients with leiomyosarcoma using publicly available data from The Cancer Genome Atlas (TCGA) cohort. Age was categorised as ≥65 years and <65 years, tumour size was categorised as ≥10 cm and <10 cm, and CD8 scores were classified into high and low groups for the top 1/3 (26 patients) and bottom 1/3 (26 patients). Multivariate analyses were performed by adding age, which generally correlates with prognosis, to factors significantly different in the univariate analysis.

b. Overall survival.

c. Metastasis-Free Survival.

b, c. Likelihood ratio test. Differences between the two groups were considered statistically significant at *p* < 0.05.

Supplementary Figure 1. Comparison of tumour-infiltrating immune cell counts in primary and lung metastasis tissues of malignant soft tissue tumours.

a. Comparison of tumour-infiltrating immune cell counts in primary and lung metastasis tissues of the entire malignant soft tissue tumour cohort (*n* = 38).

b. Comparison of tumour-infiltrating immune cell counts in primary and lung metastasis tissues of undifferentiated pleomorphic sarcoma (*n* = 9).

c. Comparison of tumour-infiltrating immune cell counts in primary and lung metastasis tissues of synovial sarcoma (*n* = 6).

a-c. Wilcoxon signed-rank test. **p* < 0.05.

Supplementary Figure 2.

Comparison of CD8^+^ cell numbers in primary and lung metastatic tissues of undifferentiated pleomorphic sarcoma (*n* = 9), synovial sarcoma (*n* = 6), myxofibrosarcoma (*n* = 3), myxoid liposarcoma (*n* = 3), dedifferentiated liposarcoma (*n* = 2), and alveolar soft part sarcoma (*n* = 2). The lines indicate that they were obtained from the same patient. Wilcoxon signed-rank tests were used to compare differences between the two groups. Differences between the two groups were considered statistically significant at *p* < 0.05.

Supplementary Figure 3.

a-d. CD8 staining at the tumour cores and tumour margins were assessed by counting the number of positive cells in five different fields using a 10× eyepiece lens and a 40× objective lens. The cut-off values for high and low infiltration at the tumour cores were 100 cells/mm^2^ and for high and low infiltration at the tumour margins were 200 cells/mm^2^. Primary tumours and lung metastases of leiomyosarcoma (LMS) were classified into four groups each according to high/low tumour core and high/low tumour margins. (*n* = 13 and *n* = 13, respectively)

a. Representative images of primary tumours of LMS in each group.

b. Representative images of lung metastases of LMS in each group.

c. Comparison of the number of infiltrating CD8^+^ T cells in tumour cores and tumour margins in primary tumours and lung metastases, respectively.

d. Comparison of margin/core CD8 ratios by violin plot in primary tumours and lung metastases, respectively.

c, d. Wilcoxon signed-rank test. **p* < 0.05.

Supplementary Figure 4.

a. Comparison of the number of tumour-infiltrating clusters of differentiation (CD)8^+^ cells in programmed death-ligand 1 (PD-L1)^+^ leiomyosarcoma and PD-L1^-^ leiomyosarcoma.

b. Representative image of PD-L1 immunohistochemical (IHC) staining of leiomyosarcoma.

c. Comparison of the number of tumour-infiltrating CD8^+^ cells in human leukocyte antigen (HLA) class I ABC^high^ leiomyosarcoma and HLA class I ABC^low^ leiomyosarcoma.

d. Representative image of HLA class I ABC IHC staining in leiomyosarcoma.

e. Flow cytometry analysis of CD8^+^ T cells isolated from whole blood collected from healthy donors.

a, c. Mann-Whitney *U*-test. Differences between the two groups were considered statistically significant at *p* < 0.05.

Supplementary Figure 5.

a-f. Analysis of the effect of epithelial cellular adhesion molecule (EPCAM) inhibition and knockdown on the migration of cluster of differentiation (CD)8^+^ T cells in the human leiomyosarcoma cell line TC616.

a, b. TC616 cells treated with EPCAM inhibitors-produced conditioned media. Tumour necrosis factor-α converting enzyme (TACE) inhibitor (TAPI), and γ-secretase inhibitor (DAPT) were used. In addition to the inhibition of EPCAM signalling by the combination of TAPI and DAPT, partial inhibition of EPCAM signalling was also performed with each drug alone. The migration of CD8^+^ T cells was evaluated and compared using a Transwell assay with conditioned medium.

c-f. TC616 cells were transfected with siEPCAM or scrambled siRNA-produced conditioned media. The migration of CD8^+^ T cells was evaluated and compared using a Transwell assay with conditioned medium.

a, e. The membrane of the Transwell insert was stained, and the number of migrated CD8^+^ T cells was evaluated. Representative microscopy images are shown.

b, f. Comparison of the numbers of migrated CD8^+^ T cells. For each membrane shown in (a) and (e), the number of stained positive cells was counted in five different fields using a 10× objective lens, and the total number was evaluated.

c. EPCAM expression in each cell line was determined by reverse transcription-quantitative polymerase chain reaction (RT-qPCR).

d. EPCAM expression in each cell line was determined by western blotting.

Data in b and f are mean ± standard deviation (SD); *n* =3. Two-trailed *t*-test. **p* < 0.05, ***p* < 0.01.

Supplementary Figure 6.

a. Venn diagram illustrating the number of genes detected by analysis of differentially expressed genes (DEGs) in epithelial cell adhesion molecule (EPCAM)-inhibited and EPCAM-knockdown human leiomyosarcoma cell line TYLMS-1 cells.

b-e. Analysis of the effect of synaptosomal-associated protein of 25 kDa (SNAP25) knockdown on cluster of differentiation (CD)8^+^ T cell migration in EPCAM knockdown human leiomyosarcoma cell line TC616. Conditioned media were prepared by transfection of siEPCAM + siSNAP25, siEPCAM, or scramble siRNA into TC616 cells. The migration of CD8^+^ T cells was evaluated and compared using a Transwell assay with conditioned medium.

b. The expression of EPCAM and SNAP25 in each cell line was determined using reverse transcription-quantitative polymerase chain reaction (RT-qPCR).

c. Expression of EPCAM and SNAP25 in each cell line was determined using western blotting.

d. The membrane of the Transwell insert was stained, and the number of migrated CD8^+^ T cells was evaluated. Representative microscopic images are presented.

e. Comparison of the number of migrated CD8^+^ T cells. In each membrane shown in (e), the number of stained positive cells was counted in five different fields using a 10× objective lens, and the total number was evaluated. Data are presented as the mean±standard deviation (SD), *n* =3. Two-trailed *t*-test. **p* < 0.05, ***p* < 0.01.

Supplementary Figure 7.

a-f. Analysis of the effect of epithelial cellular adhesion molecule (EPCAM) and synaptosomal-associated protein of 25 kDa (SNAP25) knockdown on the proliferation and migration of the human leiomyosarcoma cell line TYLMS-1 and TC616.

a. Proliferation assay of TYLMS-1. (*n* = 3)

b. Proliferation assay of TC616. (*n* = 3)

c, d. The membrane of the Transwell insert was stained, and the number of migrated tumour cells was evaluated. Representative microscopy images are shown.

c. Migration assay of TYLMS-1. (*n* = 3)

d. Migration assay of TC616. (*n* = 3)

e, f. Comparison of the numbers of migrated tumour cells. For each membrane shown in (c) and (d), the number of stained positive cells was counted in five different fields using a 10× objective lens, and the total number was evaluated.

Data in a, b, e, and f represent the mean ± standard deviation (SD). Two-trailed *t*-test. **p* < 0.05, ***p* < 0.01.
